# Comorbidity Analysis between Alzheimer’s Disease and Type 2 Diabetes Mellitus (T2DM) Based on Shared Pathways and the Role of T2DM Drugs

**DOI:** 10.3233/JAD-170440

**Published:** 2017-09-18

**Authors:** Reagon Karki, Alpha Tom Kodamullil, Martin Hofmann-Apitius

**Affiliations:** a Department of Bioinformatics, Fraunhofer Institute for Algorithms and Scientific Computing (SCAI), Schloss Birlinghoven, Sankt Augustin, Germany; b Rheinische Friedrich-Wilhelms-Universität Bonn, Bonn-Aachen International Center for IT, Bonn, Germany

**Keywords:** Alzheimer’s disease, comorbidity, disease mechanisms, disease modeling, metformin, OpenBEL, type 2 diabetes mellitus

## Abstract

**Background:**

Various studies suggest a comorbid association between Alzheimer’s disease (AD) and type 2 diabetes mellitus (T2DM) indicating that there could be shared underlying pathophysiological mechanisms.

**Objective:**

This study aims to systematically model relevant knowledge at the molecular level to find a mechanistic rationale explaining the existing comorbid association between AD and T2DM.

**Method:**

We have used a knowledge-based modeling approach to build two network models for AD and T2DM using Biological Expression Language (BEL), which is capable of capturing and representing causal and correlative relationships at both molecular and clinical levels from various knowledge resources.

**Results:**

Using comparative analysis, we have identified several putative “shared pathways”. We demonstrate, at a mechanistic level, how the insulin signaling pathway is related to other significant AD pathways such as the neurotrophin signaling pathway, PI3K/AKT signaling, MTOR signaling, and MAPK signaling and how these pathways do cross-talk with each other both in AD and T2DM. In addition, we present a mechanistic hypothesis that explains both favorable and adverse effects of the anti-diabetic drug metformin in AD.

**Conclusion:**

The two computable models introduced here provide a powerful framework to identify plausible mechanistic links shared between AD and T2DM and thereby identify targeted pathways for new therapeutics. Our approach can also be used to provide mechanistic answers to the question of why some T2DM treatments seem to increase the risk of AD.

## INTRODUCTION

Alzheimer’s disease (AD) and type 2 diabetes mellitus (T2DM) are prevalent in aging populations. In particular, AD confronts us with the challenge of finding early stage diagnostic biomarkers that can be used for prevention and treatment and may help control the progression of the disease [1, 2]. In contrast, several classes of Food and Drug Administration (FDA) approved drugs like thiazolidinediones [3], DPP4-inhibitors [4], and GLP1 receptor agonists [5] are available for the treatment of T2DM. Despite the fact that T2DM is a metabolic disorder and AD is a central nervous system disease, an increasing number of epidemiological studies suggest that there is a significant comorbid association between T2DM and AD [6, 7].

The comorbid association could be due to shared pathophysiology processes that are underlying both diseases [8, 9]. When we try to understand the putative shared pathophysiology of AD and T2DM, some important questions need to be addressed. Firstly, are the current methodologies capable and competent to provide better understanding of co-morbidity and their underlying pathophysiology processes? Secondly, how can we establish a mechanistic link between two medical conditions confined to specific regions of the body (brain for AD and liver for T2DM)? One of the major limitations of the conventional comorbidity measurement approaches is that they rely on clinical readouts and the association between these readouts is purely statistical. In order to establish mechanistic links between comorbid diseases, we systematically harvested and modeled relevant information on molecular mechanisms potentially shared by these diseases.

Another notion supporting the concept of shared mechanisms underlying comorbidity is based on the observation that the medication used for one disease could itself be a risk factor for the initiation or progression of another disease. There are many case reports about the initiation or modulation of a disease due to the usage of drugs for another indication. For example, drug-induced-parkinsonism has been observed in older patients due to the use of antipsychotic drugs such as haloperidol (HALDOL), chlorpromazine (THORAZINE) [10], thioridazine (MELLARIL) [11], trifluoperazine (STELAZINE) [12], and fluphenazine (PROLIXIN) [13]. The risk associated with antipsychotics is often dose dependent and related to dopamine D2 striatal occupancy, which is linked mechanistically to parkinsonism [14, 15].

Previous attempts to understand the comorbid association between AD and T2DM have not considered the role of dysregulated entities such as genes/proteins, SNPs, and miRNAs and impaired biological processes involved in the diseases but rather have focused on specific biological pathways of interest. Associations between biological pathways and comorbid observations are usually reported in the form of free text; whereas pathway information is commonly represented in various pathway databases. A context-specific, knowledge-based network modeling approach, however, may provide a better way to integrate all the scientific knowledge around comorbid diseases and to identify common underlying mechanisms. Motivated by the capabilities of the Biological Expression Language (BEL) [16] to construct cause and effect computable network models, we have therefore generated AD and T2DM models based on knowledge extracted from the scientific literature. The resulting mechanistic network modeling work was driven by two hypotheses: 1) Impaired pathways in T2DM increase the risk for AD, and 2) T2DM drugs increase the risk of AD. Using our mechanistic modeling approach, we have tried to unravel shared pathways possibly perturbed by a drug prescribed for one of the comorbid diseases which could be causally involved in the etiology the other comorbid disease. The models developed here not only represent a comprehensive view on shared pathways between the two diseases but also provide a means to mechanistically differentiate the effects induced by treatments and explain how it contributes to comorbidity.

## METHODS

### Data collection and model building

Firstly, SCAIView [17], a tool that allows semantic search and retrieval of articles, was used to build well-defined literature corpora. Secondly, all the articles were manually checked for their relevancy to the context of diseases. Thirdly, BEL coding experts read through articles to extract essential lines of evidence; which were subsequently encoded into BEL statements. We have considered a total of 448 articles, which were manually converted to BEL statements to build the AD model. Similarly, a total of 106 articles that explicitly focus on the shared mechanisms of T2DM with AD were considered to build the T2DM model. We have also extracted relevant information from databases after manually checking the referenced articles. In this way, two comprehensive systems biology models specific to AD and T2DM were built from mostly human-based PubMed articles and available pathway databases like KEGG and Reactome.

### Identification of common pathways between AD and T2DM

To identify shared pathways, which are enriched in our models of AD and T2DM, we have performed gene set enrichment analysis using a functional annotation tool provided by the Database for Annotation, Visualization and Integrated Discovery (DAVID) [18]. The tool enables users to retrieve a wide range of annotations, mainly GO terms, biological pathways, protein-protein interactions, and disease associations, represented by a given list of genes. The significance of each resulting annotation is determined by a *p*-value calculated by modified Fisher Exact test, where the smaller the *p*-value, the more significant is the output. We deployed the tool to functionally annotate the list of genes from our models with biological processes or pathways. Sub-graphs representing canonical pathways were extracted from the models and analyzed to identify shared edges and nodes amongst the enriched pathways.

### Investigation of the role of T2DM drugs to AD pathology

Using SCAIView, we retrieved all drugs mentioned in the literature for T2DM with the idea in mind to analyze, whether T2DM drugs have been reported to cause AD. We found 1,060 entries/drug names from 78,248 documents, which were ranked based on relative entropy [19]. We further analyzed the role of top 20 T2DM drugs based on the mechanism derived from our AD models. The aim of this analysis was to identify T2DM treatments that could be associated with the development of AD.

## RESULTS

### Causal and correlative BEL models representing mechanistic pathways in AD and T2DM

Using a literature mining approach, we selected 448 and 106 articles, which were found to contain relevant information about AD and T2DM, respectively. The AD BEL model consists of 2,004 nodes and 4,766 edges representing 3,068 BEL statements. The nodes consist of 539 proteins, 273 biological processes, 176 SNPs, 163 complexes, 140 chemical entities, 136 genes, 45 RNAs, 41 miRNAs, 23 pathologies, and 468 other entities representing translocation, transcription, and degradation processes. Similarly, in the context of T2DM, we have extracted 1,333 BEL statements to build a network comprising of 1,094 nodes and 2,414 edges. The nodes consist of 183 proteins, 146 biological processes, 327 SNPs, 48 complexes, 86 chemical entities, 92 genes, 24 RNAs, 12 miRNAs, 28 pathologies, and 148 other entities representing processes like translocation, transcription, and degradation. There are about 31 commonly impaired bio-processes (mainly: insulin resistance, insulin signaling pathway, oxidative stress, mitochondrial dysfunction, degradation of beta cells, neuron apoptosis, etc.), which are associated with both AD and T2DM. Likewise, 9 common diseases/pathologies like cardiovascular disorders, obesity, and amyloidosis were found to be common to both AD and T2DM models, which are often co-mentioned with AD and T2DM.

### Cross talk between insulin signaling pathway and other AD specific pathways with respect to AD and T2DM

In order to identify shared signaling pathways perturbed in both AD and T2DM, we have performed systematic comparisons of the two models based on gene sets (pathways) derived from the models by applying the DAVID tool. This analysis allowed us to prioritize the pathways already enriched in our models and to further extract shared mechanisms or sub-networks common to both models. Among others, we have identified insulin signaling pathway, neurotrophin signaling pathway, PI3K/AKT signaling, MTOR signaling, MAPK signaling, and microglial mediated immune responses as the top-ranked pathways. We analyzed further how these specific pathways do cross-talk to each other, potentially contributing to the comorbidity between AD and T2DM.

**Fig.1:**
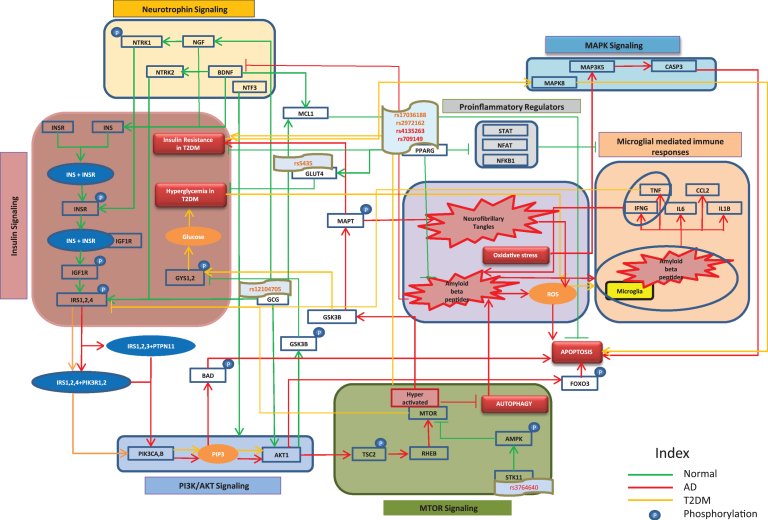
Cross talk between significant pathways in AD and T2DM. The cartoon represents interactions among entities of different signaling pathways involved in AD and T2DM. Red, orange, and green edges represent AD, T2DM, and normal conditions, respectively. Here, we depict the role of insulin signaling pathway and involvement of other pathways like PI3K/AKT signaling, MOTR signaling, MAPK signaling, and Neurotrophin signaling in AD and T2DM which give rise to characteristic features of both the disease.

As shown in Fig. 1, in normal insulin signaling pathway, insulin (INS) binds to the insulin receptor (INSR) causing a phosphorylation of INSR, thereby activating INSR to bind to insulin like growth factor 1 receptor (IGF1R). This interaction phosphorylates IGF1R to further activate insulin receptor substrate 2 (IRS2) and insulin receptor substrate 4 (IRS4). In AD, IRS2 and IRS4 interact with protein tyrosine phosphatase, non-receptor type 11 (PTPN11) and phosphoinositide-3-kinase regulatory subunit 1 (PIK3R1) to activate phosphatidylinositol-4, 5-bisphosphate 3-kinase catalytic subunit alpha (PIK3CA) which increase phosphatidylinositol (3, 4, 5)-trisphosphate (PIP3). Similar to these events in AD, PIP3 activation by insulin receptor substrates through PIK3CA and their receptors has been observed in T2DM. PIP3 increases apoptosis by increased phosphorylation of BCL2 associated agonist of cell death (BAD) and activation of forkhead box O3 (FOXO3) through AKT serine/threonine kinase 1 (AKT1) [20]. Furthermore, AKT1 hyper-activates the mechanistic target of rapamycin (MTOR) through increased activation of a Ras homologue enriched in brain (RHEB) by phosphorylating the tuberous sclerosis 2 protein (TSC2) [21]. Inactivation of MTOR promotes autophagy, thereby regulating the process of removal of amyloid-β (Aβ) from the brain [22, 23]. The presence of Aβ hinders the activity of brain derived neurotrophic factor (BDNF), which is known to regulate INS [24]. In T2DM, hyperactivated MTOR inhibits normal phosphorylation of insulin substrates, affecting insulin sensitivity and thereby increasing insulin resistance, which in turn leads to apoptosis through upregulation of mitogen-activated protein kinase 8 (MAPK8) [25]. Likewise, tumor necrosis factor (TNF) in T2DM is also responsible for abnormal serine phosphorylation of insulin substrates [26]. In normal brain, AKT1 is involved in phosphorylating glycogen synthase kinase 3β (GSK3β), restricting the ability of GSK3β to further phosphorylate microtubule associated protein tau (MAPT), which will otherwise lead to increased deposition of that protein in neurofibrillary tangles (NFTs) [27]. Moreover, it also regulates the intake of glucose by decreasing phosphorylation of glycogen synthase 1 (GYS1) and glycogen synthase 2 (GYS2) [28, 29]. We found that AKT1 is consistently downregulated in AD as well as in T2DM, which supports the fact that glucose levels are increased in blood, a condition called hyperglycemia [30]. Hyperglycemia in T2DM increases reactive oxygen species, thereby becoming detrimental to normal beta cell function. Similarly in AD, NFTs and Aβ increase oxidative stress by producing reactive oxygen species, leading to activation of microglia and inflammatory regulators [31, 32]. The increase in oxidative stress reported in AD, increases levels of mitogen-activated protein kinase kinase kinase 5 (MAP3K5), which further adds to caspase 3 (CASP3) activity resulting in apoptosis [33].

To support the above-mentioned pathways and mechanisms, we analyzed gene expression datasets (Supplementary File 1 and 2) to understand the expression patterns of the entities involved in AD and T2DM. The expressions of protein kinase AMP-activated catalytic subunits (PRKAA1 and PRKAA2) and protein kinase AMP-activated non-catalytic subunits (PRKAB2 and PRKAG3), which code for AMP-activated protein kinase (AMPK) sub-units, were found to be downregulated in AD, which is consistent with the above depicted Fig. 1. Similarly, protein kinase AMP-activated non-catalytic subunit gamma 1 (PRKAG1) and protein kinase AMP-activated non-catalytic subunit gamma 2 (PRKAG2), which also code for AMPK sub-units, were found to be downregulated in T2DM. Moreover, serine/threonine kinase 11 (STK11), a protein that activates AMPK [34], was found to be under-expressed in both AD and T2DM. Likewise, we found downregulated signal transducer and activator of transcription (STAT1 and STAT2), nuclear factors of activated T-cells (NFATC1, NFATC2, and NFATC3), and nuclear factor kappa B subunit 1 (NFKB1) to be associated with AD conditions, all of which are pro-inflammatory regulators associated with microglia-mediated immune response. Correspondingly, in the context of T2DM, downregulation of STAT1 and nuclear factor of activated T-cells 4 (NFATC4) were observed. The expressions of neurotrophic receptor tyrosine kinases (NTRK1 and NTRK2) and neurotrophin 3 (NTF3), which regulate insulin-signaling pathway, were found to be down regulated in AD, while only NTRK2 was downregulated in T2DM. However, we did not find any significant signals regarding the expression of glucagon (GCG), solute carrier family 2 member 4 (SLC2A4), peroxisome proliferator activated receptor gamma (PPARG), and BCL2 family apoptosis regulator (MCL1) from the data sets we analyzed. Nevertheless, we could identify certain SNPs that are associated with T2DM and AD in patient cohorts through literature. We identified SNP rs12104705 to be associated with GCG [35], SNP rs5435 with SLC2A4 [36], and SNPs rs1801282 and rs1805192 with PPARG [37, 38]. These genetic variants may contribute to the perturbation of normal functions of these genes/proteins in the disease state. A dedicated mechanistic analysis and more independent cohort studies are needed to prove the functional role of these genetic variants in causing the comorbidity. One such analysis identifying STK11 genetic variant rs3764640 in regulating autophagy has been depicted by Kodamullil et al. [39].

### Comorbidity analysis based on use of drugs

Using our in-house text mining tool, SCAIView, we retrieved FDA-approved drugs from AD and T2DM to understand the perspective of comorbidity in the context of drugs. Among the 5 approved AD drugs, we found that only tacrine has some effects on T2DM [40, 41]. In contrast, 20 approved T2DM drugs have already been investigated for repurposing for AD (Supplementary File 3) targeting mainly PPARG, AMPK, GCG, and leptin (LEP). In the following sections, we briefly discuss positive and negative effects of various drug targets taking into account relevant studies (mostly human based and few animal based) and further elaborate on the effects of metformin in AD.

PPARG functions by regulating SLC2A4, a protein that plays a vital role in T2DM as it enhances transportation and absorption of glucose [42]. Since a type of brain-specific-diabetes is observed in AD [43], targeting PPARG in AD has been widely considered. Moreover, PPARG is capable of inhibiting pro-inflammatory regulators, which are responsible for microglial activation [44]. The over-activation of microglia has been shown to lead to deposition of Aβ peptides through excess release of inflammatory factors in AD [45]. The second common target, AMPK, improves glucose metabolism and insulin sensitivity in T2DM, a much-needed activity in the normal brain [46, 47]. For this reason, T2DM drugs targeting AMPK are considered as repurposing candidates for AD. The other common targets, GCG and LEP, are also interesting targets in T2DM. Although they are not directly involved in the insulin signaling pathway, they can have an effect on AD pathophysiology mechanisms. Inducing glucose lowering effects in T2DM, GCG is able to improve synaptogenesis and neurogenesis, inhibit depositions of Aβ and microglial activation in AD [48]. Likewise, LEP activation has been shown to reduce enzymatic activity of beta-secretase 1 (BACE1) and phosphorylation of MAPT [49].

However, some contradictions reporting the findings that T2DM drugs may increase the risk of AD keep the chances of repurposing T2DM drugs at bay. Rosiglitazone, an agonist of PPARG, has a very low blood-brain barrier penetration [50]. Thus, induced insulin sensitivity in the brain might not be good enough to regulate normal insulin signaling. On the other hand, as it effectively sensitizes peripheral tissues to insulin, the levels of blood insulin are remarkably decreased, thereby reducing insulin levels in brain. This is assumed to promote neuronal insulin resistance over time [51]. Metformin, a T2DM drug, is known to upregulate expression of BACE1 and increase deposition of Aβ [52]. Furthermore, sitagliptin has been reported to increase MAPT phosphorylation, eventually leading to NFTs [53]. In this regard, it can be concluded that T2DM drugs amplify the risk to develop AD or at least serve as a risk factor in AD. This highlights the need of shifting research works from repurposing T2DM drugs in AD to not-well-known possible influences of T2DM drugs in developing AD. Above all, it is of utmost importance to understand the mechanisms modified by T2DM drugs that will eventually lead to AD.

### Identification of the role of metformin in AD using disease models

To identify the potential role of drugs in causing or at least modulating the risk of comorbidity between diseases, we have performed a mechanistic analysis of the top T2DM drug targets in the context of AD. The search of drugs related to T2DM using SCAIView indicated that metformin belongs to the drugs with highest relevance (based on relative entropy scores) [54]. Metformin is a FDA-approved biguanide anti-hyperglycemic agent used for the treatment of T2DM. The mechanism of action of metformin is understood to reduce blood glucose levels by decreasing glucose production in the liver, reducing intestinal absorption of glucose and improving insulin sensitivity by increasing uptake and utilization of glucose in the peripheral regions of the body [55]. Modeling the drug-target-pathway context of metformin resulted in a complex pattern: we were able to explain, at a mechanistic level, the discrepancy of epidemiological observations that are linked to effects of drug treatment. These mechanistic explanations are in sharp contradiction to previously suggested opportunities of repurposing metformin in AD. We can, however, also reconstruct the most likely mechanistic explanation for the beneficial effects of metformin. It remains to be shown, how far genetic variation (SNPs) and epigenetics effects account for the differences observed in epidemiological studies.

### Putative beneficial effects

**Fig.2:**
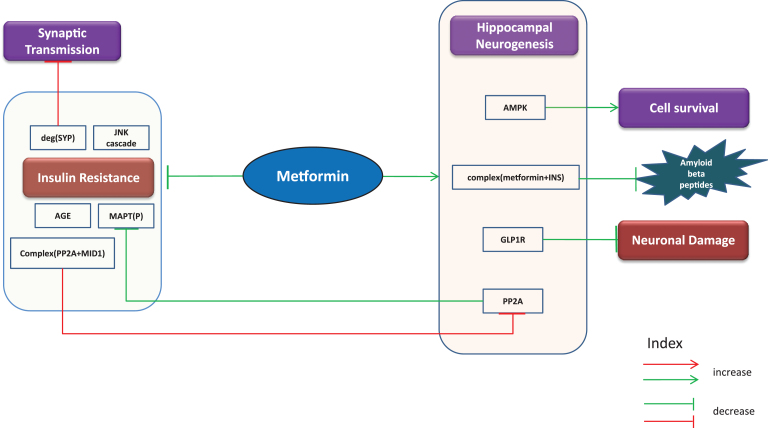
Putative beneficial effects. Putative beneficial effects of metformin represented as cartoon diagram: The green edges refer to effects induced by metformin or events in normal conditions while the red edges indicate events in diseased state. The capability of metformin to reduce insulin resistance and neuronal damage, promote cell survival and hippocampal neurogenesis and inhibit AGEs, JNK cascade, and phosphorylation of MAPT provides us with the opportunity to repurpose metformin for AD.

Since AD is frequently accompanied by insulin resistance [56, 57], metformin is sought to improve insulin sensitivity in AD patients. It is also capable of inhibiting MAPT phosphorylation through increased activity of PP2A as evident from a study from Kickstein et al. [58]. The authors explain this mechanism by reporting the finding that metformin in fact interrupts the binding of PP2A with MID1/α4 complex, a protein-protein interaction involved in degradation of PP2A. Hence, it is likely to prevent formation of NFTs and reduce progression of AD [58]. Similarly, it inhibits neuronal damage via upregulation of glucagon like peptide 1 receptor (GLP1R), a glucagon receptor [59]. The combined use of metformin and INS is reported to reduce the aggregation of Aβ [52]. Furthermore, the drug is known to inhibit the JNK cascade, formation of advanced glycation end products (AGE) and protect against degradation of synaptophysin (SYP), a protein involved in synaptic transmission [60, 61]. In addition, AMPK’s ability to promote cell survival is surged by metformin [62]. Through our gene expression analysis of mice samples (Supplementary File 1), we observed that PP2As and AMPKs increased with metformin treatment while SYP was found to be decreased. A simple cartoon representation of beneficial effects of metformin is shown below in Fig. 2.

### Putative harmful effects

**Fig.3:**
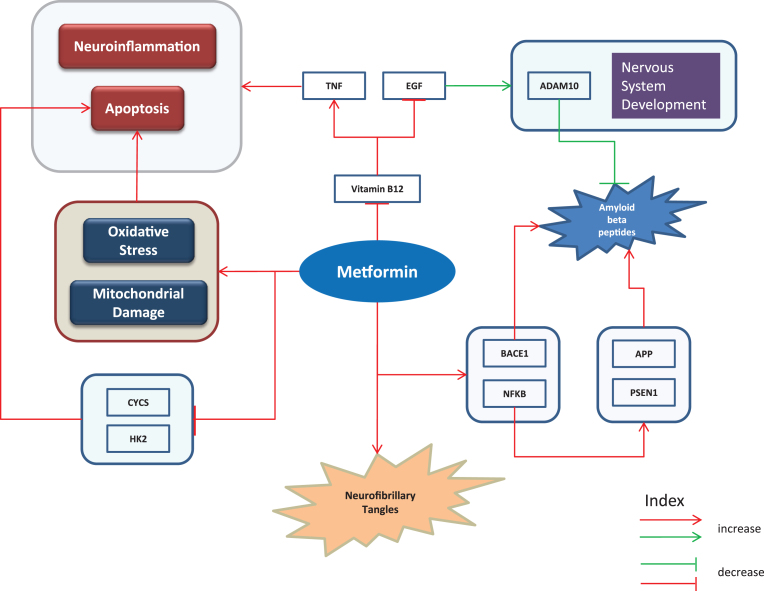
Putative harmful effects. Harmful effects of metformin represented as cartoon diagram: The red edges represent effects of metformin or events observed in diseased state while the green edges refer to events in normal conditions. Metformin’s use has been reported to contribute to characteristic features of AD such as apoptosis, neuroinflammation, neurofibrillary tangle formation, and aggregated amyloid-β questioning the suggested opportunities seen in metformin as a repurposible drug.

The following “chain of causation” (Fig. 3) provides a mechanistic explanation for the observed harmful effects of metformin. We assume that “modifiers” (e.g., the genetic makeup of individual or epigenetic modifications) outside of our models may contribute to the overall decision making process that results in either beneficial or harmful effects.

Metformin adds to deposition of Aβ by increasing transcriptional activity of BACE1 [52]. Picone et al. [63] report the findings that it contributes in accumulation of Aβ through NFKB1 activation which further upregulates PSEN1 and APP. The same study reveals that treatment with metformin increased oxidative stress and mitochondrial damage and decreased expressions of Cytochrome C (CYCS) and Hexokinase 2 (HK2). As a result of these effects of metformin, cell death was observed [63]. In addition, it promotes insoluble tau aggregation as reported by Barini et al. suggesting that it could possibly increase the risk of tauopathy among metformin treated diabetic patients [64]. The drug also reduces activity of vitamin B12 which causes reduced epidermal growth factor (EGF) and increased TNF [65], the latter of which is often associated with apoptosis and neuroinflammation. In normal conditions, EGF has a positive effect on nervous system development and levels of A Disintegrin And Metalloproteinase domain-containing protein 10 (ADAM10), a protein known to reduce Aβ aggregation [66]. The expression patterns of the aforementioned genes (Supplementary File 1) were analyzed to understand their concordances with literature. The upregulated expressions of BACE1, PSEN1, and APP identified from gene expression analyses of mice and human samples are consistent with the literature findings. In contrast, expression of EGF in mice was observed to be downregulated.

## DISCUSSION

A large number of epidemiological, preclinical, and pathophysiology studies indicate that AD and T2DM share cellular and molecular mechanisms. The classical approaches in measuring comorbidity that are based on clinical readouts, patient data, and electronic health records cannot reason over the dysfunctional molecular activity or the impaired biological pathway involved in the diseased state. On the contrary, deciphering comorbidity at a mechanistic level could well explain the outcomes of clinical readouts and patient examinations establishing a link between proteomic/genomic and phenotypic aspects of diseases. However, in this study we do not attempt to cover this proposal. Since there are no established studies aimed at explaining comorbidity based on shared mechanisms, we believe that understanding the co-morbid mechanisms between complex diseases can be dealt with systems biology approaches like integrative modeling. We followed a knowledge-driven modeling approach, which served as a rationale to infer the mechanistic background of comorbidity association between AD and T2DM. Modeling using BEL bears a high “explanatory” potential; although we do not necessarily discover new knowledge, we bring information into context and are able to reconstruct mechanisms. As we are aware of the publication bias, we have therefore done model-validation through available data (gene expression profiles) as the key to identify contradictions or concordances between formalized knowledge and patterns in data.

The synopsis of mechanisms relevant for T2DM and AD reveals that there is crosstalk among important pathways that play either a role in T2DM or AD and are thus candidates for shared pathways possibly involved in the observed comorbidity. The results of this study demonstrate that encoding relevant knowledge into causal relationship models confers enhanced interpretation power that is well-suited for comorbidity analysis. Our results provide additional support to previously suggested comorbid associations between AD and T2DM. We have shown at a mechanistic level how entities involved in insulin signaling, PI3K/AKT signaling, MTOR signaling, neurotrophin signaling, and microglial-mediated immune responses interact and potentially contribute to the manifestation of characteristic features of both AD and T2DM. Since there are a few inconsistencies in the gene expression data (both humans and mice) to support the key interactions depicted in this paper, integration of genetic variants into the models may add to the explanatory power of the models and support the notion of candidate comorbid mechanisms. Furthermore, depicting the AD features induced by metformin, we hypothesize that drug treatment itself could contribute to the comorbidity between AD and T2DM. A study aimed at understanding the progression of AD by comparing metformin-treated-T2DM patients with other T2DM patients treated with other drugs is needed to validate the effect. This emphasizes the need to reconsider the prescription of drugs if there is any evidence of comorbid disease associated with any drug.

It is clear from this work that BEL based network modeling approaches bear great potential to help us to identify shared mechanisms between two diseases. However, as new knowledge is being generated and communicated all the time, we need a continuous update of the models in order to unravel new mechanisms and to take into account additional factors that may contribute to comorbidity. Additionally, as complex diseases like AD as well as T2DM progress with time, there is also a need to integrate the time dependent cascade of events, which is not currently dealt with by BEL modeling. Our hope is that this sort of analysis will allow us to identify new drug repurposing candidates based on the common mechanism between diseases. Based on our analysis, we see a need to re-evaluate the role of existing drugs because besides having positive effects against a particular disease, they might also be involved in progression of another disease. What needs to be addressed in the future is the definition of the role of genetic variants and epigenetic modifications in order to generate a comprehensive picture of the mechanisms underlying comorbidity of diseases together with the time dependences. Given the identification of candidate mechanisms for comorbidity between AD and T2DM, we propose additional experiments around these pathways to find common targets between AD and T2DM, which could pave way for new therapeutic developments that take shared mechanisms into account.

## Supplementary Material

Supplementary File 1

Supplementary File 2

Supplementary File 3
