# Supplementary material for: Comorbidity Analysis between Alzheimer’s Disease and Type 2 Diabetes Mellitus (T2DM) Based on Shared Pathways and the Role of T2DM Drugs
Source: J Alzheimers Dis. 2017 Sep 18;60(2):721–31. doi: 10.3233/JAD-170440 (PMC5611890; doi:10.3233/JAD-170440)
Supplement: Supplementary File 2 [file jad-60-jad170440-s002.docx]

| **Genes** | **Expression in AD** | **Expression in T2DM** |
| --- | --- | --- |
| **AKT1** |  |  |
| **BAD** |  |  |
| **BDNF** |  | NA |
| **CASP3** |  | NA |
| **CCL2** |  |  |
| **FOXO3** |  |  |
| **GCG** | NA | NA |
| **GSK3B** |  | NA |
| **GYS1** | NA | NA |
| **GYS2** |  | NA |
| **IFNG** |  |  |
| **IGF1R** |  |  |
| **IL1B** |  |  |
| **IL6** | NA | NA |
| **INS** |  | NA |
| **INSR** |  |  |
| **IRS1** |  |  |
| **IRS2** |  | NA |
| **IRS4** | NA | NA |
| **MAP3K5** |  |  |
| **MAPK8** |  |  |
| **MAPT** |  |  |
| **MCL1** |  |  |
| **MTOR** |  | NA |
| **NFATC1** |  |  |
| **NFATC2** |  | NA |
| **NFATC3** |  | NA |
| **NFATC4** |  |  |
| **NFATC5** | NA | NA |
| **NFKB1** |  | NA |
| **NGF** |  | NA |
| **NTF3** |  | NA |
| **NTRK1** |  | NA |
| **NTRK2** |  |  |
| **PIK3CA** |  | NA |
| **PIK3CB** |  |  |
| **PIK3R1** |  |  |
| **PIK3R2** |  |  |
| **PPARG** |  | NA |
| **PRKAA1** |  | NA |
| **PRKAA2** |  | NA |
| **PRKAB1** |  | NA |
| **PRKAB2** |  | NA |
| **PRKAG1** |  |  |
| **PRKAG2** |  |  |
| **PRKAG3** |  | NA |
| **PTPN11** |  |  |
| **RHEB** |  |  |
| **SLC2A4** |  | NA |
| **STAT1** |  |  |
| **STAT2** |  |  |
| **STK11** |  |  |
| **TNF** | NA | NA |
| **TSC2** |  |  |

Note: This table shows the expression patterns of genes from various GEO datasets. The green arrows represent upregulated expression and red arrows represent down regulation. **NA** refers to unavailable or insignificant expression patterns. This table is a summary of Supplementary Table 1 (Sheet names: AD and T2DM) where the expression values of genes from different datasets are shown. This table is a part of the result section titled: **Cross talk between insulin signaling pathway and other AD specific pathways with respect to AD and T2DM.**
